# Scarless excision of an insertion sequence in the OmpK36 promoter restores meropenem susceptibility in a non-carbapenemase-producing *Klebsiella pneumoniae*

**DOI:** 10.1080/22221751.2025.2503922

**Published:** 2025-05-09

**Authors:** Yingying Du, Tong Liu, Yuanzhi Gong, Yinghua Yuan, Yunlou Zhu, Min Hao, Yuhao Liu, Sheng Wang

**Affiliations:** a Intensive Care Medical Center, Tongji Hospital, School of Medicine, Tongji University, Shanghai, People’s Republic of China; b Department of Critical Care Medicine, Shanghai Tenth People’s Hospital, School of Medicine, Tongji University, Shanghai, People’s Republic of China; c Department of Critical Care Medicine, Zhongshan Hospital, Fudan University, Shanghai, People’s Republic of China; d Department of Clinical Microbiology, Shanghai Tenth People’s Hospital, School of Medicine, Tongji University, Shanghai, People’s Republic of China; e Institute of Antibiotics, Huashan Hospital, Fudan University, Shanghai, People’s Republic of China

**Keywords:** Non-carbapenemase-producing *Klebsiella pneumoniae*, meropenem resistance, OmpK36 deficiency, promoter, insertion sequence

## Abstract

Carbapenem-resistant *Klebsiella pneumoniae* (CRKP) poses a significant global health challenge due to its limited treatment options and high mortality rates. Meanwhile, the prevalence of non-carbapenemase-producing CRKP (NC-CRKP) strains is increasing, but their resistance mechanisms remain less understood compared to those of carbapenemase-producing CRKP (CP-CRKP). In this study, KP-469, an NC-CRKP strain, was found to lack the major porins OmpK35 and Ompk36 but possessed OmpK37, coexisting with ESBL resistance genes CTX-M and SHV. Membrane porin coding sequence alignment revealed a minor deletion in Ompk35 and a 768 bp insertion sequence in the promoter region (IS-PR) of Ompk36, located between the -10 region and the ribosome-binding site (RBS). In the KO-469 strain with scarless excision of IS-PR and the constructed pHSG396-promoter-Ompk36 strain that incorporated wild-type Ompk36 promoter into KP-469, the transcription levels of Ompk36 were significantly higher than that in KP-469 strain, and His-tag antibody quantification further confirmed the regular expression of Ompk36 in KO-469. These results demonstrated that IS-PR markedly reduced the transcriptional and translational efficiency of Ompk36 in the KP-469 strain, leading to decreased permeability to meropenem. Moreover, the restored susceptibility to meropenem in the KO-469 strain was validated by *in vitro* antimicrobial susceptibility tests and an *in vivo* intraperitoneal infection model constructed in neutrophil-depleted mice. The novel carbapenem resistance mechanism of NC-CRKP caused by the insertion sequence in the OmpK36 promoter will facilitate the development of antibacterial regimens for treating NC-CRKP infections.

## Introduction

*Klebsiella pneumoniae* (*K. pneumoniae*) is a crucial Gram-negative bacterial pathogen that often causes community or hospital-acquired infections in the respiratory tract, abdominal cavity, urinary tract, nervous system, and other regions [1]. The China Antimicrobial Surveillance Network has ranked *K. pneumoniae* second among all clinical isolates, with its proportion increasing from 9.5% in 2005 to 14% in 2022 [2]. Moreover, the prevalence of carbapenem-resistant *K. pneumoniae* (CRKP) has shown a significant upward trend, from 2.9% in 2005 to 25.0% in 2018 [2], as supported by data from the China Antimicrobial Resistance Surveillance System [3]. Non-carbapenemase-producing CRKP (NC-CRKP) refers to the strains that lack carbapenemase hydrolytic activity but are resistant to carbapenem. Prolonged and increased exposure to carbapenem antibiotics has increased the prevalence of these pathogens, as recent studies have suggested that NC-CRKP comprises 50% or more of CRKP isolates [4,5]. Moreover, the disease burden caused by NC-CRKP should not be ignored. In an Italian study, the death rate of NC-CRKP-infected patients (37.9%) is similar to that of CP-CRKP-infected patients (38.9%) [6]. Another recent study in Malaysia found that the all-cause in-hospital mortality rate was 52.2% in NC-CRKP-infected patients, suggesting that NC-CRKP may be more virulent than CP-CRKP [7].

The resistance of NC-CRKP to carbapenem antibiotics is attributed to a combination of decreased outer membrane permeability and/or increased expression of efflux pumps, frequently in conjunction with the production of extended-spectrum β-lactamases (ESBLs) or AmpC cephalosporinases [8]. In *K. pneumoniae*, OmpK35 and Ompk36 are the primary non-specific porins, and their dysfunction is frequently associated with carbapenem resistance. Reduced porin permeability, particularly involving these porins, plays a pivotal role in developing resistance in NC-CRKP [9,10]. Previous studies have linked reduced porin permeability to aberrant amino acid sequences and decreased porin expression. Frameshift mutations or premature stop codons, resulting from inserting foreign fragments and/ or deletion of small DNA segments, have been shown to lead to abnormal amino acid sequences in membrane porins [11,12]. Amino acid substitutions in key domains of porins can also disrupt the charge distribution in the constriction region of the loops, thereby altering the channel’s permeability to antibiotics [13]. In addition to porin modification, the XylS–AraC transcriptional regulators (MarA, SoxS, Rob, RamA, and CpxA) and small noncoding RNAs (sRNAs), such as micF, micA, micC, and RybB, have been shown to cause carbapenem resistance through the post-transcriptional regulation of porin expression [14].

However, research on reduced porin permeability has primarily focused on abnormalities in the coding region of porins, the role of promoters in regulating porin expression has received limited attention. This study aims to elucidate the influence of promoter sequences on porin expression and their involvement in antibiotic resistance in NC-CRKP.

## Materials and methods

### Selection of strains

Between January and December of 2023, all *K. pneumoniae* clinical isolates collected from sputum, urine, blood, and other body fluid samples of patients admitted to Shanghai Tenth People’s Hospital, Tongji University, were screened for ESBL production, followed by the NG-Test® CARBA5 assay (FOSUNDAGNOSTICS, China) to confirm carbapenemases production. A total of 98 *K. pneumoniae* isolates producing ESBLs without carbapenemase activity were included in this study (Supporting Information, Figure A).

### Drug sensitivity test and efflux pump inhibition test

All the included strains were tested *in vitro* for antimicrobial susceptibility using the VITEK®AST2 plate (bioMérieux, French). The results were interpreted according to the Clinical and Laboratory Standards Institute M100-31st edition breakpoints for all agents except for polymyxin and tigecycline. ESBL production was assessed using the Kirby–Bauer method, and efflux activity was inhibited by adding carbonyl cyanide m-chlorophenylhydrazone (CCCP) at a final concentration of 25 mg/L. A reduction in the minimum inhibitory concentration (MIC) by more than 4-fold following the addition of CCCP suggests the overexpression of efflux pumps in the isolates [15].

### Determination of resistance and porin genes in non-carbapenemase-producing K. pneumoniae

Multiplex PCR was performed to detect ESBL resistance genes (*bla_CTX-M_*, *bla_SHV_*, *bla_DHA_*, *bla_TEM_,* and *bla_CYM_*) and OMP genes (*OmpK35*, *OmpK36*, and *OmpK37*). The PCR primers used are listed in Table S1. Reactions were performed using a Multiplex PCR kit (Vazyme, Nanjing, China), following the manufacturer’s instructions. The PCR products were purified and sequenced, and the DNA sequences were compared with the reported nucleotide sequences in GenBank.

### Detection of outer membrane porin in KP-469

OMPs were isolated following a streamlined protocol adapted from Carlone et al. [16]. Following extraction, OMPs were separated using sodium dodecyl sulfate-polyacrylamide gel (12%) electrophoresis, and protein bands were visualized using Coomassie Blue R-250 staining. ATCC 13883 strain was used as the reference control.

### Whole-genome sequencing (WGS) of KP-469 strain

WGS was conducted using the Illumina HiSeq X-10 platform (Illumina, San Diego, CA, USA), generating 150 bp paired-end reads. After quality control, clean reads were assembled using SOAPdenovo V2. PacBio RS II single-molecule real-time sequencing was integrated with Illumina sequencing to obtain the complete KP-469 genome. The combined data were assembled using Unicycler (version 0.4.7) and annotated using Prokka (version 1.14.6) with default parameters. Online alignment of OmpK35, OmpK36, and the promoter, transcription factors MarA/SoxS/Rob, RamA, and CpxA sequences in KP-469 were performed using NCBI. Genome completeness was evaluated using the BUSCO database (version 5.2.2) [17].

### Quantitative reverse transcription PCR of KP-469 strain

The transcriptional levels of membrane porins OmpK35/OmpK36, XylS–AraC transcription regulators (RamA and CpxA), and sRNAs (RybB and MicC) were determined using quantitative real-time PCR (RT-qPCR) in strain KP-469. Each reaction was conducted in triplicate, and fold changes in gene expression were calculated as previously described. ATCC 13883 served as the reference *K. pneumoniae* strain, while rpoB was the endogenous reference gene. All RT-qPCR primers are listed in Table S1.

### Ompk35/OmpK36 gene deficiency and mutations in non-carbapenemase-producing K. pneumoniae from the NCBI database

In this study, the genome data of 1152 non-carbapenemase-producing *K. pneumoniae* strains were retrieved from the NCBI database. The coding sequences of porin genes were systematically compared to evaluate the frequencies of mutations and deletions. Abnormalities in the promoter regions were identified for further investigation. The flowchart of the standardized procedure for non-carbapenemase-producing *K. pneumoniae* strains collection in NCBI is shown in the Supporting Information (Figure B).

### The construction of KO-469 with the scarless excision of IS in the OmpK36 promoter region

The suicide plasmid pRE112 was used to knock out the insertion sequence in the promoter of the porin Ompk36 in KP-469. Primer pairs designed for the targeted gene knockout are listed in Table S1, and relevant plasmids and bacterial strains are provided in Table S2. The detailed protocol for the gene knockout is documented in experimental section C of Supporting Information.

### The expression of recombinant OmpK36-His-tagged fusion protein in KO-469

The pBeloBAC11 plasmid was used to produce recombinant His-tagged OmpK36 fusion protein. Primers designed for plasmid construction are listed in Table S1, while the utilized plasmids and bacterial strains are provided in Table S2. The protocol for vector construction and Ompk36 protein expression is outlined in experimental section D of the Supporting Information.

### Complementing OmpK36 loss with wild-type promoter

The plasmid pHSG39-promoter-OmpK36 was constructed to complement OmpK36 loss in KP469, and the plasmid pHSG396-promoter-IS-OmpK36, in which the promoter with IS-PR was used as the control. Both plasmids were transformed into the recipient strain KP469, and successfully complemented strains were selected using plates containing antibiotics and verified by PCR sequencing. The primers used for complementation are listed in Table S1. RNA was extracted from the complementary strains for RT-qPCR to detect the effects of the two types of promoters on the transcription level of OmpK36.

### Bacterial viability assay

To assess the antibacterial effect of meropenem on KO-469 strains *in vitro*, a 0.5 McFarland suspension of freshly cultured KP-469 and KO-469 strains was diluted 100-fold, and meropenem was added to reach a final concentration of 1 mg/L. After 2 h of incubation at 37 °C, samples were centrifuged (3000*g*, 2 min), and the supernatant was discarded. DOMA/PI dye was added to a final concentration of 1×, followed by 20 min of incubation at 37°C in the dark. The samples were then placed on a slide and observed under a Leica fluorescence microscope using a 40× objective lens (excitation, 503/530 nm; emission, 535/617 nm).

### Bacterial loads in tissues

The therapeutic efficacy of meropenem against KO-469 strain infection *in vivo* was systematically evaluated *in vivo* was assessed in a mouse model of abdominal infection. Cyclophosphamide was first administrated intraperitoneally in the adult male C57BL/6 mice at doses of 150 and 100 mg/kg on Days 0 and 3, respectively, to develop a neutrophil-depleted model and prevent the inactivation effect of neutrophils on bacterial strains [18]. On Day 4, the mice were randomly divided into KP-469 and KO-469 groups, and an intraperitoneal injection of 10^6^ colony-forming units (CFUs) of KP-469 or KO-469 was administered. Subsequently, 4 h after bacterial inoculation, all mice were administered 50 mg/kg meropenem *via* intraperitoneal injection, with subsequent doses administered every 12 h. The mice were euthanized with an overdose of pentobarbital sodium after 1 d of meropenem treatment; the lungs, spleen, kidneys, and liver were collected immediately and homogenized in 1 mL of PBS. Bacterial burden in each tissue was quantified *via* enumeration of CFUs following serial dilution of tissue grinding plating on MH agar. A 2-log reduction in the colony count was used as the criterion to assess the therapeutic efficacy of meropenem. The tissues were fixed in paraformaldehyde and embedded in paraffin. Serial 3 μm sections were stained with haematoxylin and eosin (HE) to visualize tissue alterations.

### Statistical analysis

Statistical analyses were performed using GraphPad Prism v9.5.1. Porin transcript levels between the KO-469 and KP-469 strains were compared using one-way analysis of variance (ANOVA), followed by Dunnett’s multiple comparison test. Two-way ANOVA was used to analyse tissue bacterial load, and Dunnett’s multiple comparison test was used to determine significance. The transcript levels of porins in ESBL-producing isolates were compared using Student’s *t*-test, and statistical significance was determined using the Holm–Sidak method (*P* < .05).

## Results

### The discovery of an NC-CRKP strain (KP-469)

Among the 98 NC-*K. pneumoniae* isolates, antimicrobial susceptibility testing revealed that 97 strains (98.9%) were susceptible to meropenem, whereas a single strain (KP-469) demonstrated resistance to meropenem, with a MIC of 4 mg/L. However, this strain remained susceptible to imipenem (MIC ≤ 0.5 mg/L) (Table S3). The Kirby–Bauer disk diffusion assay with clavulanic acid-supplemented disks identified 64 bacterial isolates exhibiting phenotypic characteristics that were consistent with ESBL production. All ESBL-producing isolates carried at least one ESBL resistance gene, but none possessed AmpC-type resistance genes. Besides, Ompk35/36/37 porins were detected in 77 isolates, and 6 strains coexisted with ESBL resistance genes (Table 1 and Table S4). Porin deficiency is more prevalent in ESBL-producing isolates compared with non-ESBL-producing isolates (Table 2). KP-469 was the only isolate resistant to meropenem, characterized by OmpK37 coexisting with the ESBLs resistance genes *bla_CTX-M_* and *bla_SHV_* but lacking Ompk35 and Ompk36 (Figure S1).

**Table 1.** The screening of β-lactamase and OMP genes in the collected *K. pneumoniae* isolates.

β-Lactamase resistance genes	Number of isolates	Outer membrane porins	Number of isolates
CTX-M	9	OmpK35	3
SHV	29	OmpK36	1
CTX-M and SHV	40	OmpK37	1
CTX-M and TEM	2	OmpK35, OmpK36 and OmpK37	77
CTX-M, SHV, and TEM	11	OmpK35 and OmpK36	3
SHV and TEM	7	OmpK35 and OmpK37	4
AmpC	Deficiency	OmpK36 and OmpK37	3
		Deficiency	6

**Table 2.** The impact of OmpK35/OmpK36 deficiency on MICs of MEM in non-carbapenemase-producing *K. pneumoniae*.

	MEM MIC ≤ 0.25 mg/L			MEM MIC = 0.5 mg/L			MEM MIC = 1 mg/L			MEM MIC > 4 mg/L		
	OmpK35 deficiency	OmpK36 deficiency	–	OmpK35 deficiency	OmpK36 deficiency	–	OmpK35 deficiency	OmpK36 deficiency	–	OmpK35 deficiency	OmpK36 deficiency	–
ESBLs+	1	3	6	0	1	1	0	0	0	0	0	1
ESBLs−	0	0	0	0	0	0	0	0	0	0	0	0

Note: MIC, MEM and “–” represent minimal inhibitory concentration, meropenem and the simultaneous deficiency of OmpK35 and OmpK36, respectively.

### IS-PR is a potentially novel insertion mode causing OmpK36 deficiency

**Figure 1.**
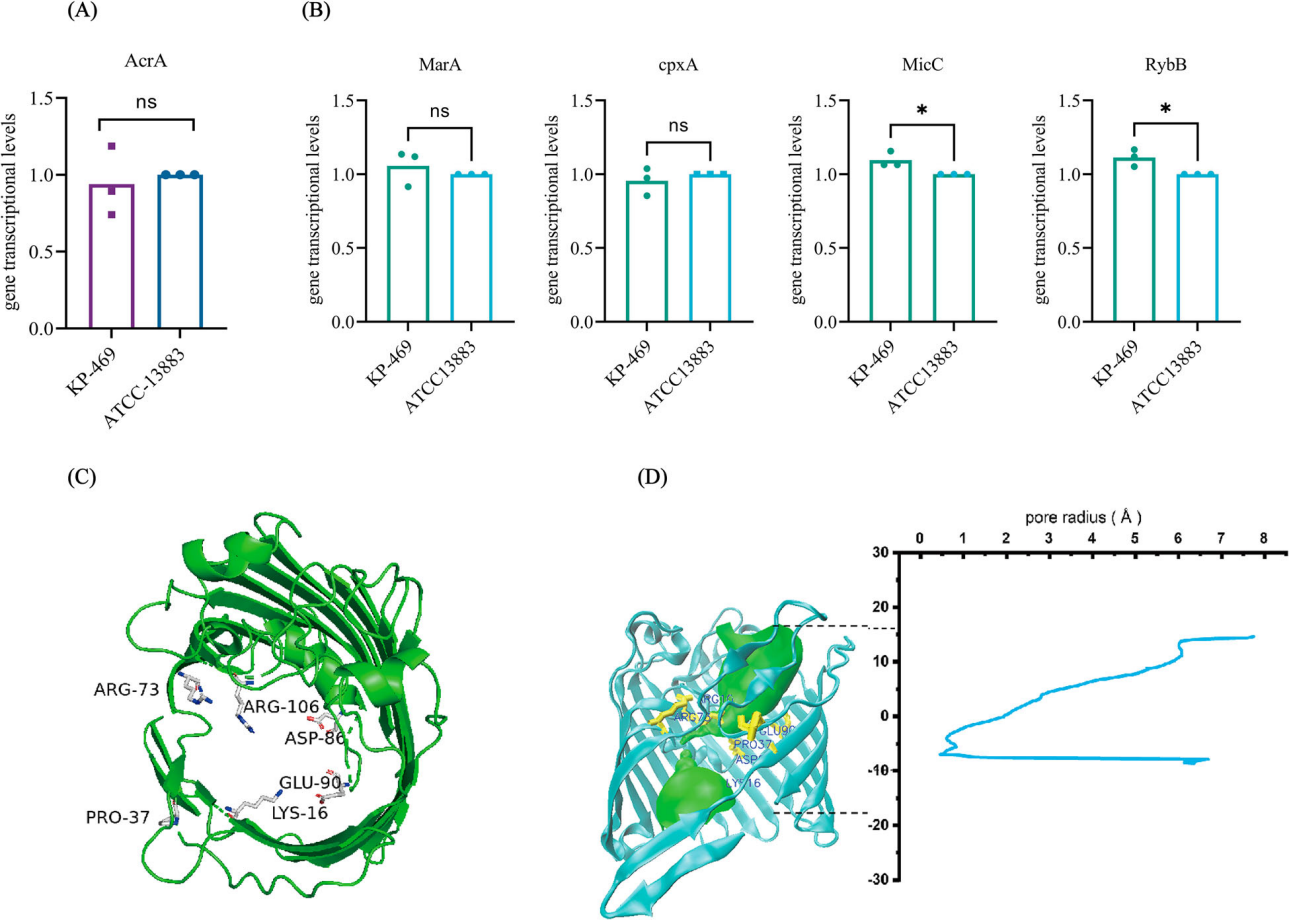
OmpK36 deficiency in KP-469 was not related to porin channel structure alteration, efflux pump overexpression, or post-transcriptional regulation. (A) No overexpression of efflux pump gene AcrA was found in KP-469. (B) RT-qPCR showed no overexpression of regulatory transcription factors (RamA and cpxA) and small RNAs (sRNAs) (micC and RybB) in KP-469. (C) Amino acid residues in the OmpK36 contraction region. (D) Predicted amino acid structure and pore radius analysis of OmpK36 in KP-469.

**Figure 2.**
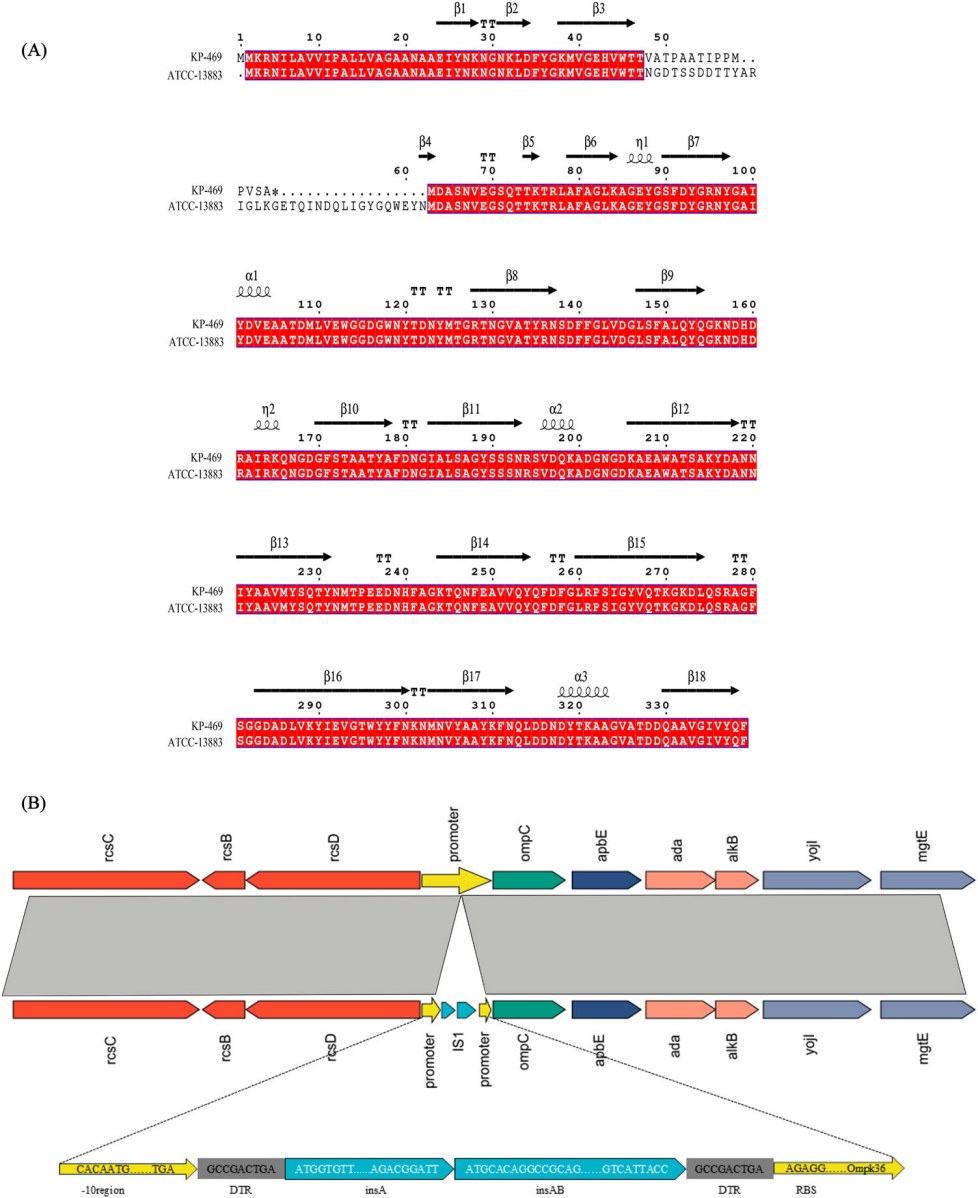
The mechanisms of Ompk35/Ompk36 deficiency in KP-469. (A) The amino acid sequence of OmpK35 indicated a 53-base deletion upstream of the OmpK35 coding sequence. (B) Insertion sequence (IS) within the OmpK36 promoter located between the -10 region and the ribosome-binding site.

The efflux pump inhibition assay showed that the addition of CCCP (25 mg/L) did not affect the MIC of meropenem and imipenem (Table 3), and RT-qPCR did not detect increased expression of efflux pump genes (Figure 1(A)). Thus, meropenem resistance in KP-469 was not associated with the overexpression of efflux pumps but was likely attributed to reduced outer membrane permeability. This hypothesis was supported by RT-qPCR findings, showing significantly lower transcriptional levels of OmpK35 and Ompk36 in KP-469 than those in other meropenem-susceptible ESBL-producing *K. pneumoniae* strains (Figure S1 C, D). Because the reduced transcription of porins correlates with their deficiency, we conducted an in-depth analysis to elucidate the mechanism underlying porin deficiency. Initial comprehensive sequence alignment analyses of the transcription factors and small RNAs (sRNAs) revealed no mutations in the transcription factor sequences. The transcriptional levels of the transcription factors (MarA, CpxA) and sRNAs (micC, RybB) showed no significant increase (Figure 1(B)). Moreover, the amino acid structure prediction of porins revealed that the amino acids within the constriction zone of OmpK36 had no amino acid substitutions, and the diameter of its porin pores was within normal parameters (Figure 1(C,D)). Finally, the coding sequence based on WGS data showed that OmpK35 had a 53-base-pair deletion starting at the 118th base pair, leading to a premature stop codon (Figure 2(A)). An unexpected discovery was that the OmpK36 promoter showed a 768-bp insertion sequence between the -10 region and the ribosome-binding site (RBS) (Figure 2(B)), suggesting that the loss of OmpK36 is attributed to IS-PR.

**Table 3.** Susceptibility profiles of *K. pneumoniae* strains with CCCP.

Strains	MIC (mg/L)			
	MEM	MEM+CCCP	IPM	IPM+CCCP
ATCC13883	0.125	0.125	0.125	0.125
KP-469	4	4	0.5	0.5
KO-469	1	1	0.5	0.5
KP-469/pHSG396-IS-OmpK36	4	4	0.5	0.5
KP-469/pHSG396-OmpK36	–	–	0.5	0.5
KP-469/pHSG396	4	4	0.5	0.5

Note: MIC: minimum inhibitory concentration; MEM: meropenem; IMP: imipenem; CCCP: carbonyl cyanide *m*-chlorophenylhydrazone.

As summarized in Tables S5–S7, OmpK36 was detected in 265 (23%) of the 1152 non-carbapenemase-producing *K. pneumoniae* isolates screened from the NCBI GenBank database and OmpK36 synonymous mutations had a cumulative mutation frequency of 690 (86.5%). For sequence variation, TTGGAT (63.4%, 156/246) and TCCATT (34.1%, 84/246) were the predominant sequence types in the -35 region, while two main sequence types, CACAATG (63.4%, 156/246) and CCAGGGC (34.1%, 84/246), were identified in the -10 region (Table 4). However, no screened strains had upstream aberrations beyond the -35 region, and no abnormal insertion sequences were found between the -10 region and the RBS, indicating that IS-PR is a novel insertion mode in the promoter region of OmpK36 in non-carbapenemase-producing *K. pneumoniae*.

**Table 4.** Promoter sequences of NC-CRKP in the NCBI database.

-35 region	Numbers	-10 region	Numbers
TTGGAT	156	CACAATG	156
TCCATT	84	CCAGGGC	84
ATCCAT	3	ACCAGGG	3
CATTAA	1	AGGGCGA	1
TAAGCT	1	CGGCCTT	1
GAATGA	1	GAAGTCG	1

Note: NC-CRKP: non-carbapenemase-producing *K. pneumoniae*.

### IS-PR inhibited the transcription and translation of OmpK36 in KP-469

**Figure 3.**
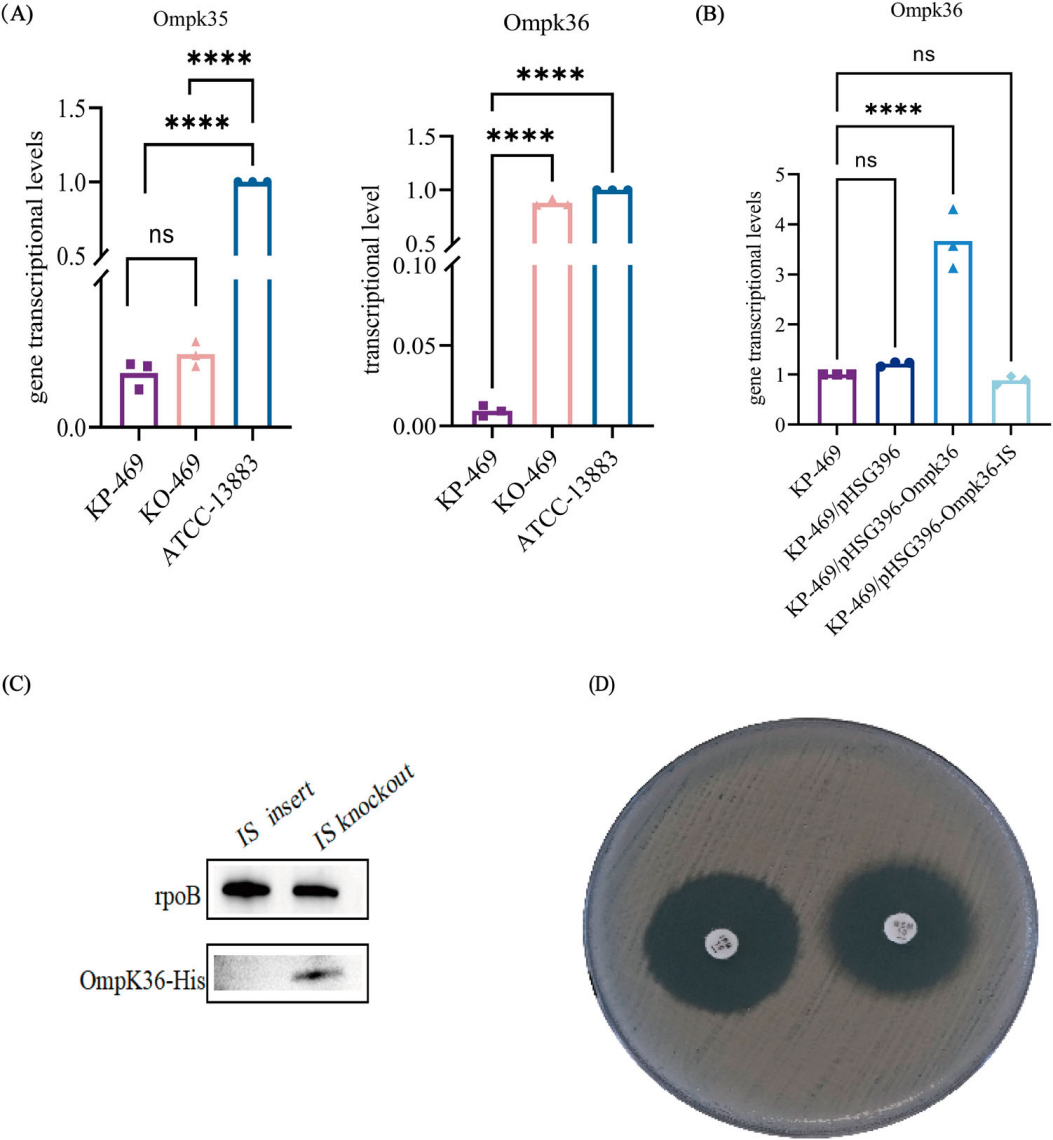
The IS-PR in the Ompk36 promoter region inhibited the transcription and translation of Ompk36 in the KP-469 strain. (A) IS-PR knockout almost completely restored the regular transcription of OmpK36 in the KO-469 strain. (B) Transcription levels of OmpK36 were tested after introducing different types of pHSG396 recombinant plasmids into KP-469. In the pHSG396-promoter-OmpK36 strain, which incorporated the wild-type OmpK36 promoter into KP-469, the transcript levels of OmpK36 were significantly higher than those of KP-469, ****P < .0001. (C) Western blot analysis confirmed that the insertion of an IS-PR element within the OmpK36 promoter region significantly impaired the expression of the OmpK36-His fusion protein. (D) The susceptibility to meropenem was restored in the KO-469 strain, as determined by the disk diffusion test.

The effect of IS-PR on the transcription level of OmpK36 was first assessed in KO-469, an IS-PR scarless excision strain. RT-qPCR analysis showed that the transcription level of OmpK36 in KO-469 was more than 199 times higher than that in KP-469 (Figure 3(A), *P* < .01).

To further validate the action of IS-PR on OmpK36 transcription, two complementary strains were constructed: pHSG396-promoter-OmpK36 with the wild-type OmpK36 promoter and pHSG396-promoter-IS-OmpK36 with the OmpK36 promoter containing IS-PR. RT-qPCR assay revealed that the transcription level of OmpK36 in the pHSG396-Ompk36 complementary strain was significantly higher than that in KP-469, whereas the transcription level in the pHSG396-IS-OmpK36 complementary strain was almost the same as that in KP-469 (Figure 3(B)). Therefore, IS-PR blocked the transcription of OmpK36.

OmpK36-His fusion protein approach was employed to determine whether diminished transcription of Ompk36 results in reduced protein expression. Western blot analysis demonstrated the absence of OmpK36-His protein in the BL21 strain harbouring an insertion sequence within the OmpK36 promoter region, whereas the appearance of an OmpK36 band verified the regular expression of the wild promoter in the BL21 strain (Figure 3(C), Figure S2). These findings demonstrate that IS-PR effectively inhibited OmpK36 expression by obstructing its translation. Furthermore, the scarless excision of IS-PR reinstated the OmpK36 promoter to its wild-type configuration, thereby facilitating the normal expression of OmpK36.

### In vitro validation of IS-PR scarless excision to restore the susceptibility of meropenem

**Figure 4.**
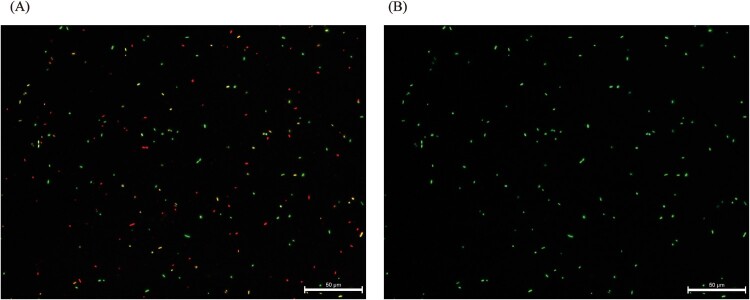
Scarless excision of IS-PR in the OmpK36 promoter region restored meropenem-sensitive phenotype *in vitro*. (A) KO-469 strain restored the meropenem-sensitive phenotype. (B) KP-469 strain showed a meropenem-resistant phenotype.

The Kirby–Bauer method (Figure S3, Figure 3(D)) revealed that the inhibition zone diameter was 27 mm for meropenem in the KO-469 strain, and the E-test™ gradient strip further confirmed that the insertion sequence knockout strain KO-469 restored susceptibility to meropenem. Moreover, after exposure to 1 mg/L (1× MIC) meropenem for 2 h, the KO-469 strain exhibited a significantly decreased viability under fluorescence microscopy, whereas the KP-469 strain had an extremely high survival rate under the same conditions (Figure 4).

### In vivo validation of IS-PR scarless excision to restore the susceptibility of meropenem

**Figure 5.**
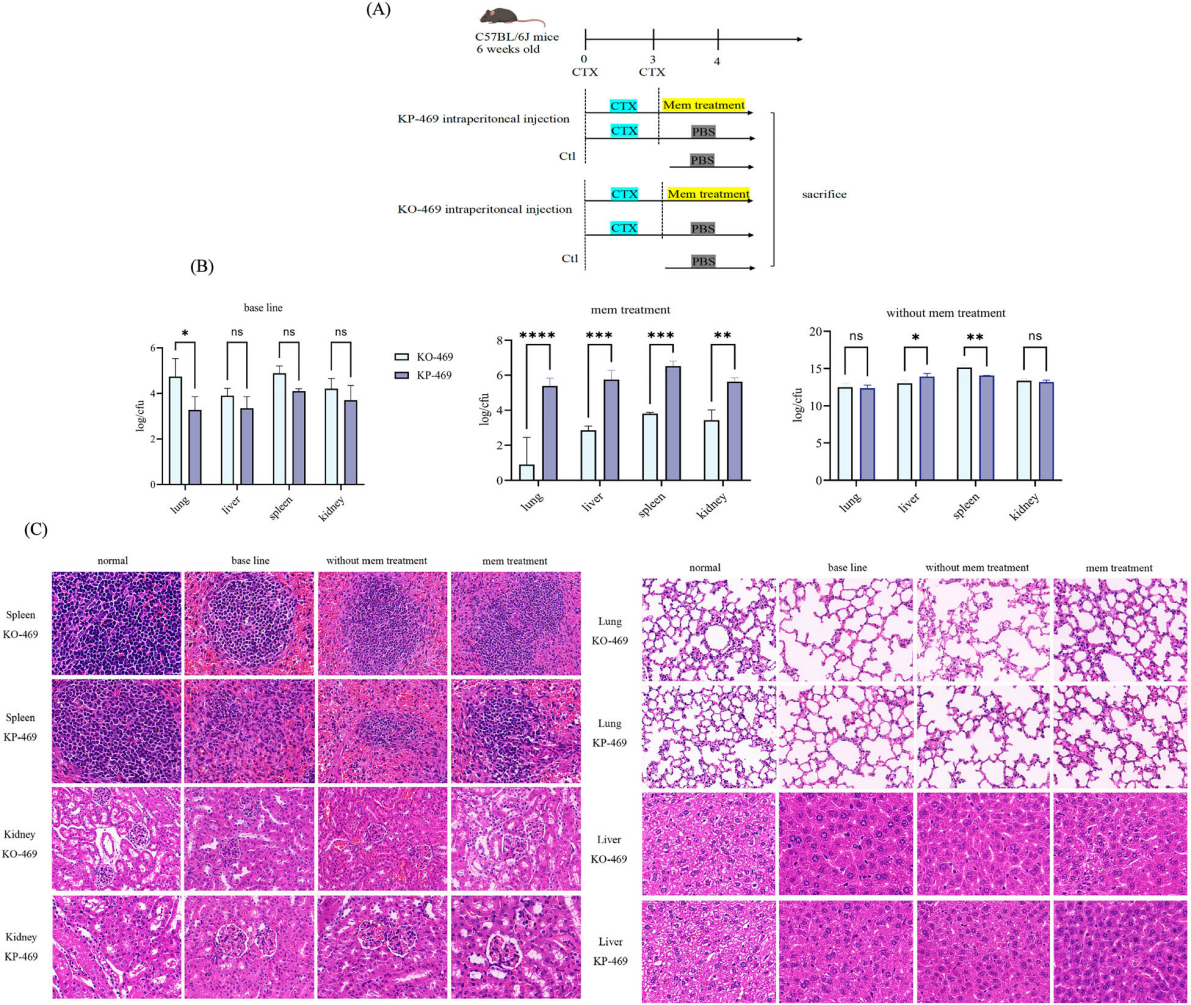
Scarless excision of IS-PR in the OmpK36 promoter region restored meropenem susceptibility *in vivo*. (A) Illustrated research protocol in mouse abdominal infection model. (B) Meropenem significantly reduced the bacterial loads in KO-469-infected mice compared to those in KP-469-infected mice. (C) Haematoxylin and eosin staining showed severe tissue damage caused by KP-469 infected mice, whereas tissue damage in KO-469 infected mice was significantly alleviated.

A neutropenic mouse model was established, as described in Figure 5(A). The presence of bacterial colonies and significantly increased levels of inflammatory factors in the tissues confirmed the successful establishment of an abdominal infection model in neutrophil-depleted mice (Figure S4, Table S8). In KO-469 infected mice, treatment with meropenem significantly reduced the lung bacterial load, manifested as a 3-fold decrease in CFU relative to the baseline inoculum (Figure 5(B)). The bacterial loads in the liver, kidneys, and spleen were also significantly lower than the baseline values. In KP-469 infected mice, however, the bacterial load in all tissues did not decrease substantially, and some even exceeded the initial inoculation dose (Figure 5(B)). Furthermore, HE staining revealed structural damage to the alveolar septa, accompanied by neutrophil infiltration within the alveolar walls in KP-469 infected mice (Figure 5(C)), and similar histopathological changes were observed in the liver, spleen, and kidneys. However, such pathological manifestations were absent in KO-469 infected mice (Figure 5(C)), indicating the restoration of meropenem efficacy *in vivo*.

## Discussion

In this study, we investigated the mechanism underlying meropenem resistance in KP-469, an NC-CRKP strain characterized by a 53-bp deletion in the coding sequence of OmpK35 and a 768-bp insertion sequence in the OmpK36 promoter. NC-CRKP is attributed to mechanisms of reduced outer membrane permeability and/or overexpression of efflux pumps, often in conjunction with the presence of ESBLs or AmpC β-lactamases [8]. However, the efflux pump inhibition and RT-qPCR assays excluded the overexpression of efflux pump genes, implying that meropenem resistance in KP-469 cells was not related to efflux pump overexpression. WGS analysis demonstrated that KP-469 harboured *bla_CTX-M_* and *bla_SHV_*, accompanied by abnormalities in OmpK35 and OmpK36. These findings suggest that the loss of OmpK35 and Ompk36 plays a pivotal role in the emergence of carbapenem resistance in strain KP-469, consistent with previous findings showing that most patients with NC-CRKP developed carbapenemase resistance to porin deficiency [7]. As the principal non-specific porins in *K. pneumoniae*, OmpK35/OmpK36 possesses high permeability to molecules < 600 Da via passive diffusion, the primary route for hydrophilic antibiotics to enter bacterial cells [19,20]. Thus, OmpK35/OmpK36 inactivation may significantly affect the influx of antibiotics, reduce their concentration in the bacterial inner membrane, and contribute to the development of antibiotic resistance. Among the 1152 *K. pneumoniae* isolates harbouring only ESBL resistance genes screened from the NCBI GenBank database, OmpK36 was detected in only 23% of the isolates, while OmpK36 had a cumulative mutation frequency of 86.5%. High defect rates of OmpK35/OmpK36 in NC-CRKP strains have also been reported in previous studies conducted in Malaysia (46.3%) and Taiwan (94.9%) [7,21]. Porin modification is attributed to changes in porin expression or channel structure [22]. In the KP-469 strain, no mutations were detected in OmpK36; however, we found that Ompk35 had a 53-bp deletion starting from the 118th base pair in KP-469. Such OmpK35 genetic variations have been reported to cause premature stop codons and are increasingly prevalent among clinical isolates [9,11]. OmpK35 deficiency appears to cause carbapenem resistance in KP-469; however, the contribution of OmpK36 deletion to carbapenem resistance in KPC-2-producing *K. pneumoniae* was more significant than that of OmpK35 deficiency. The latter can further amplify the effect of OmpK36 mutations on carbapenem resistance [23,24]; therefore, elucidating the mechanisms underlying OmpK36 deficiency remains important.

Porin modifications can also arise from structural alterations in the porin channel mediated by amino acid substitutions in loop 3, thereby altering charge distribution at these key sites and reducing the diffusion of antibiotics [23,25]. However, crystal structure analysis of OmpK36 in KP-469 revealed no evidence of amino acid substitutions in loop3. Transcription factors RamA, MarA, SoxS, and Rob of the EnvZ-OmpR two-component system can inhibit transcription by directly binding to the promoter region of porins or exerting post-transcriptional regulation through the overexpression of small RNAs (sRNAs), reducing porin expression [14].

Nevertheless, the RT-qPCR results indicated that these transcription factors were not overexpressed in KP-469. Therefore, the decrease in OmpK36 expression in this strain was not related to the negative regulatory effect of the EnvZ-OmpR two-component system. Comprehensive coding sequence alignment revealed that the OmpK36 promoter has a 768-bp IS-PR between the -10 region and the RBS. Moreover, the transcription and translation levels of OmpK36 in the KO-469 and pHSG396-promoter-OmpK36 strains were significantly higher than those in KP-469, indicating that IS1 in the OmpK36 promoter resulted in OmpK36 deficiency in KP-469. Previous studies have shown that strains with the dual knockout of OmpK35 and OmpK36 caused a 256-fold increase in the MIC of ertapenem in *K. pneumoniae* strains, suggesting that porin loss alone can lead to carbapenem resistance [9]. This viewpoint was supported by our *in vitro* and *in vivo* studies, showing that the KO-469 strains regained susceptibility to meropenem solely by restoring the expression of OmpK36.

Insertion sequence (IS) is the simplest form of intracellular mobile genetic elements, widely present in bacterial genomes. Each standard IS unit typically consists of one or two open reading frames encoding transposases to catalyse DNA excision/IS insertion, flanked by terminal inverted repeats [26]. The most common mechanism by which IS induces bacterial resistance is the interruption of gene expression [27]. For example, the interruption of the Ompk36 gene by IS caused the loss of porin expression and increased resistance to cefoxitin in *K. pneumoniae* [28]. IS1, IS5, IS10R, IS10, IS26, ISEc68, and IS903 have also been reported to cause Ompk36 deficiency by inserting into the encoding region of the Ompk36 gene in multidrug-resistant *K. pneumoniae*, including NC-CRKP [11,28,29]. Similar to other IS family members, IS1 exhibits high transposase activity and broad target site recognition capabilities. With the assistance of inverted repeat sequences, IS1 can complete the recognition, cleavage, and insertion reactions at the Ompk36 target sequence. Although these insertion sequences differ in target site selection and transposase structure, their insertion into the coding region of the Ompk36 gene consistently results in abnormal amino acid sequences, leading to a uniform resistant phenotype [30].

In our study, the IS1 sequence was inserted into the OmpK36 promoter region between the -10 region and the RBS. Furthermore, by comparing the OmpK36 encoding and promoter regions of the 1152 non-carbapenemase-producing *K. pneumoniae* strains screened from the NCBI database, no strains had upstream aberrations beyond the -35 region and no ISs were found between the -10 region and the RBS, suggesting that IS-PR between region -10 and the RBS is a novel insertion mode that causes OmpK36 deficiency in NC-CRKP.

In this study, we did not investigate how IS-PR transposition occurs in KP-469 or how IS-PR is inserted into the OmpK36 promoter. IS transposition can be induced by environmental signals, such as radiation, oxidative stress, and high temperatures. Sub-inhibitory concentrations of antibiotics promote the transposition of IS elements [27,29]; these factors may contribute to IS-PR transposition, particularly the frequent occurrence of insufficient antibiotic dosages in clinical practice. A recent study showed that the interaction between conjugative plasmids and ISs mediates the horizontal transfer of antimicrobial resistance genes [31]; therefore, conjugative plasmids might insert IS-PR into OmpK36. In addition, we did not determine whether OmpK35 or OmpK36 deficiency was more important for carbapenem resistance in NC-CRKP; therefore, further studies remain warranted.

## Conclusion

The continuous increase in NC-CRKP infection rate urgently requires the clarification of its antibiotic resistance mechanism, which is essential for clinicians to formulate optimal antibacterial regimens. This study employed genomic, molecular, and translational approaches to uncover a novel mechanism of meropenem resistance in KP-469, driven by an IS positioned between the -10 region and the RBS of the Ompk36 promoter. Our findings demonstrated that carbapenem resistance in KP-469 was attributed to Ompk36 deficiency, which was caused by the IS-PR in the Ompk36 promoter region.

## Ethics approval and informed consent

The Ethics Committee of Shanghai Tenth People’s Hospital approved the incorporation of the clinical isolates collected in this study into the *in vitro* susceptibility trials under the ethics approval code SHSY-IEC-4.1/18-74/01. This study strictly aligned with the principles stated in the Declaration of Helsinki.

## Supplementary Material

Supporting information clean revision.docx

Supplementary materials.xlsx

Figure S1.tif

FigureS4.tif

Figure S3.tif

Figure S2.tif

## Data Availability

The genome sequence of KP-469 has been deposited in GenBank under the accession number PRJNA590579.
