# Supplementary material for: Scarless excision of an insertion sequence in the OmpK36 promoter restores meropenem susceptibility in a non-carbapenemase-producing Klebsiella pneumoniae
Source: Emerg Microbes Infect. 2025 May 9;14(1):2503922. doi: 10.1080/22221751.2025.2503922 (PMC12086927; doi:10.1080/22221751.2025.2503922)
Supplement: Supporting information clean revision.docx [file TEMI_A_2503922_SM7311.docx]

**Supporting Information**

**Scarless excision of an insertion sequence in the OmpK36 promoter restores meropenem susceptibility in a** **non-carbapenemase-producing *Klebsiella pneumoniae***

Yingying Du ^a, b #^; TongLiu ^a, c #^; Yuanzhi Gong ^a, b^; Yinghua Yuan ^d^; Yunlou Zhu ^b^; Min Hao ^e^*; Yuhao Liu ^b*^; Sheng Wang ^a, b *^

^a^ Intensive Care Medical Center, Tongji Hospital, School of Medicine, Tongji University, Shanghai 200065, China

^b^ Department of Critical Care Medicine, Shanghai Tenth People’s Hospital, School of Medicine, Tongji University, Shanghai 200072, China

^c^ Department of Critical Care Medicine, Zhongshan Hospital, Fudan University, Shanghai 200032, China

^d^ Department of Clinical Microbiology, Shanghai Tenth People’s Hospital, School of Medicine, Tongji University, Shanghai 200072, China

^e^ Institute of Antibiotics, Huashan Hospital, Fudan University, Shanghai 200040, China

*Corresponding authors: Sheng Wang: [wangsheng@tongji.edu.cn](mailto:wangsheng@tongji.edu.cn).

**Table of Contents**

1. **Experimental section**
2. Standardized collection procedure for non-carbapenemase-producing *K. pneumonia*e strains from clinical specimens.
3. Genomic screening of non-carbapenemase-producing *K. pneumoniae* in the NCBI GenBank database.
4. The construction of KO-469 with the scarless excision of the insertion sequence in the OmpK36 promoter region.
5. Expression analysis of recombinant OmpK36 His-tagged fusion protein.
6. The crystal structure analysis of OmpK36 in the KP-469 strain.
7. **Results section**

**Figure S1** OmpK35/OmpK36 deficiency mediated carbapenem resistance in the KP-469 strain.

**Figure S2** IS-PR resulted in OmpK36 deficiency by translational regulation.

**Figure S3** Both Kirby-Bauer method and E-test strips confirmed the restoration of carbapenem antibiotics susceptibility in KO-469.

**Figure S4** Successful construction of abdominal infection model in the neutrophil-depleted mice.

1. **References**

**1. Experimental section**

**(A)** **Standardized collection procedure for non-carbapenemase-producing *Klebsiella pneumoniae* strains from clinical specimens.**

In this study, we isolated a meropenem-resistant *K. pneumoniae* strain (KP-469) from a critically ill patient with a bloodstream infection admitted to the Intensive Care Unit at Shanghai Tenth People’s Hospital. Antimicrobial susceptibility testing revealed that KP-469 is resistant to meropenem. However, routine carbapenemase screening by the NG-Test® CARBA5 assay (Fosun Diagnostics, China) did not find the production of carbapenemases, and molecular characterization by PCR amplification further confirmed the lack of carbapenemase resistance genes. Thus, from January 2023 to December 2023, we conducted a year-long collection of all *K. pneumoniae* clinical isolates at Shanghai Tenth People’s Hospital to investigate the potential alternative resistance mechanisms.

All collected *K. pneumoniae* strains were screened for extended-spectrum beta-lactamase (ESBL) production, followed by the NG-Test® CARBA5 assay to confirm whether carbapenemases were produced. Once the *K. pneumoniae* strains were verified to produce ESBLs without carbapenemase activity, bacterial DNA was extracted, and the primers listed in **Supplementary material Table S1** were used for PCR amplification of ESBL-associated resistance genes (*bla_CTX-M_*, *bla_SHV_,* *bla_TEM_*, *bla_DHA_*, *bla_CYM_*) and porin genes (OmpK35, OmpK36, OmpK37). Amplified products were sequenced and analyzed through comparative alignment with reference sequences to characterize genetic features. This study included 98 non-carbapenemase-producing *K. pneumoniae* isolates, including KP-469, and the detailed collection process is illustrated in **Figure A**.

**
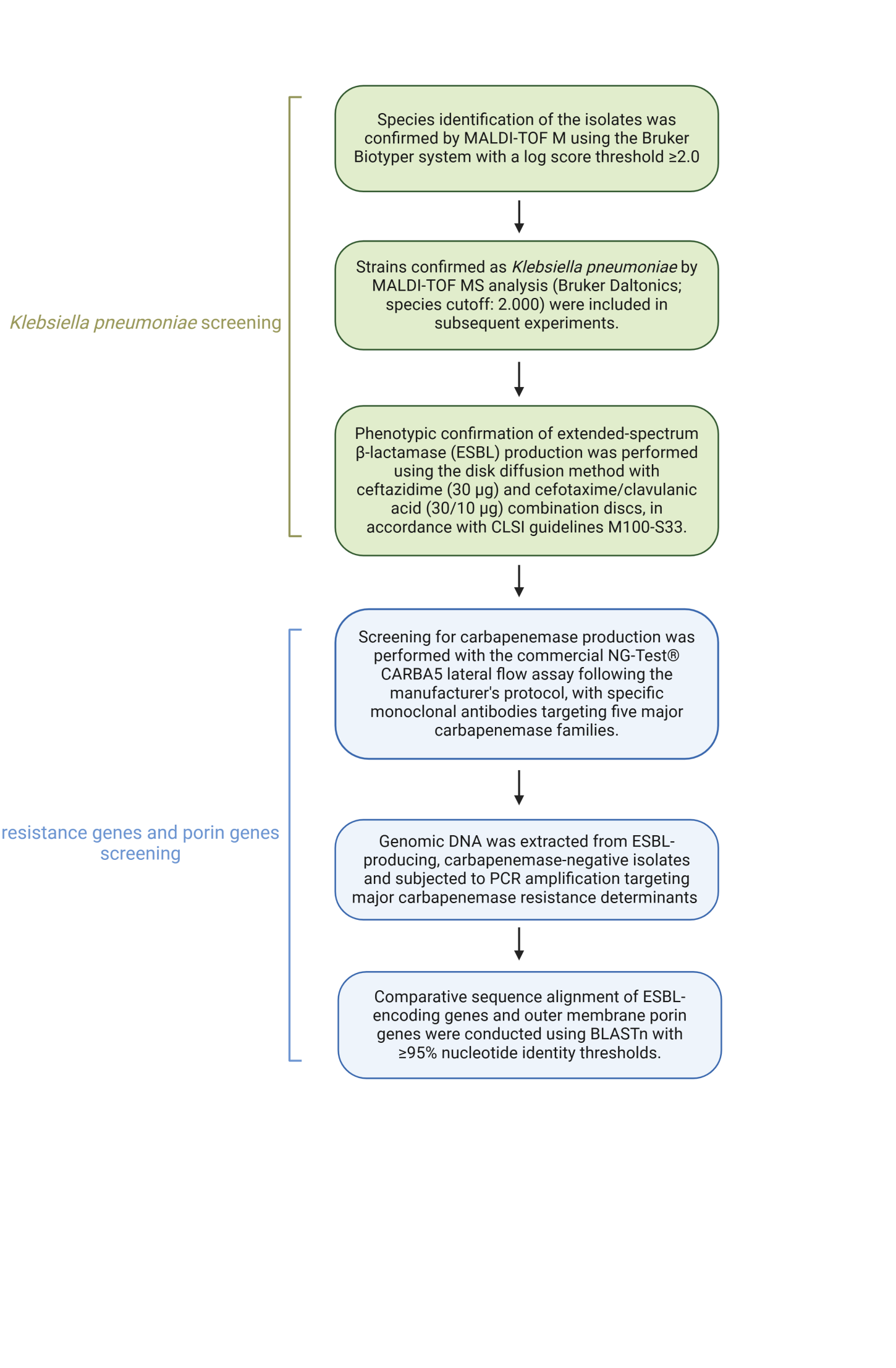
**

**Figure A** The standardized collection procedure for non-carbapenemase-producing *K. pneumoniae* strains.

**(B) Genomic screening of non-carbapenemase-producing *Klebsiella pneumoniae* in the NCBI GenBank database.**

*K. pneumoniae* strains with the whole genome data were retrieved from the NCBI database, but the strains carrying carbapenemase resistance genes were excluded. A total of 1152 non-carbapenemase-producing *K. pneumoniae* strains meeting the above screening criteria were subsequently analyzed.


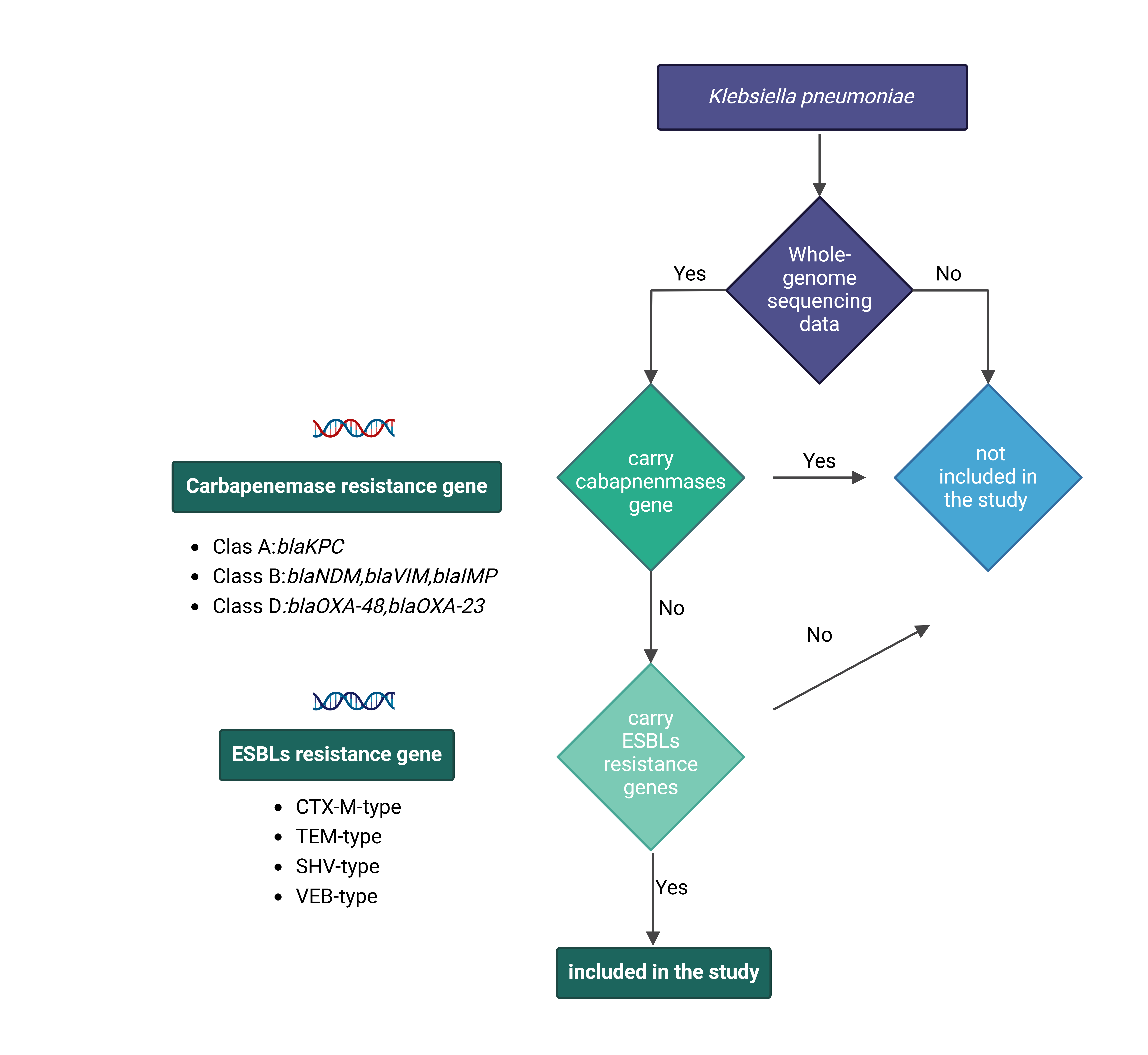
The coding sequences of OmpK35 and Ompk36 were analyzed to determine the frequency of porin deletions and frameshift mutations. Promoter regions within 1,000 bp upstream of the Ompk36 start codon were examined, focusing on their lengths and sequence variations, particularly in the conserved -35 and -10 regions. **Figure B** illustrates the standardized procedure for non-carbapenemase-producing *Klebsiella pneumoniae* strain collection from the NCBI GenBank database.

**Figure B** Flowchart of the standardized procedure for non-carbapenemase-producing *Klebsiella strains* collection from the NCBI GenBank database.

**(C) The construction of KO-469 with the scarless excision of the insertion sequence in the OmpK36 promoter region**


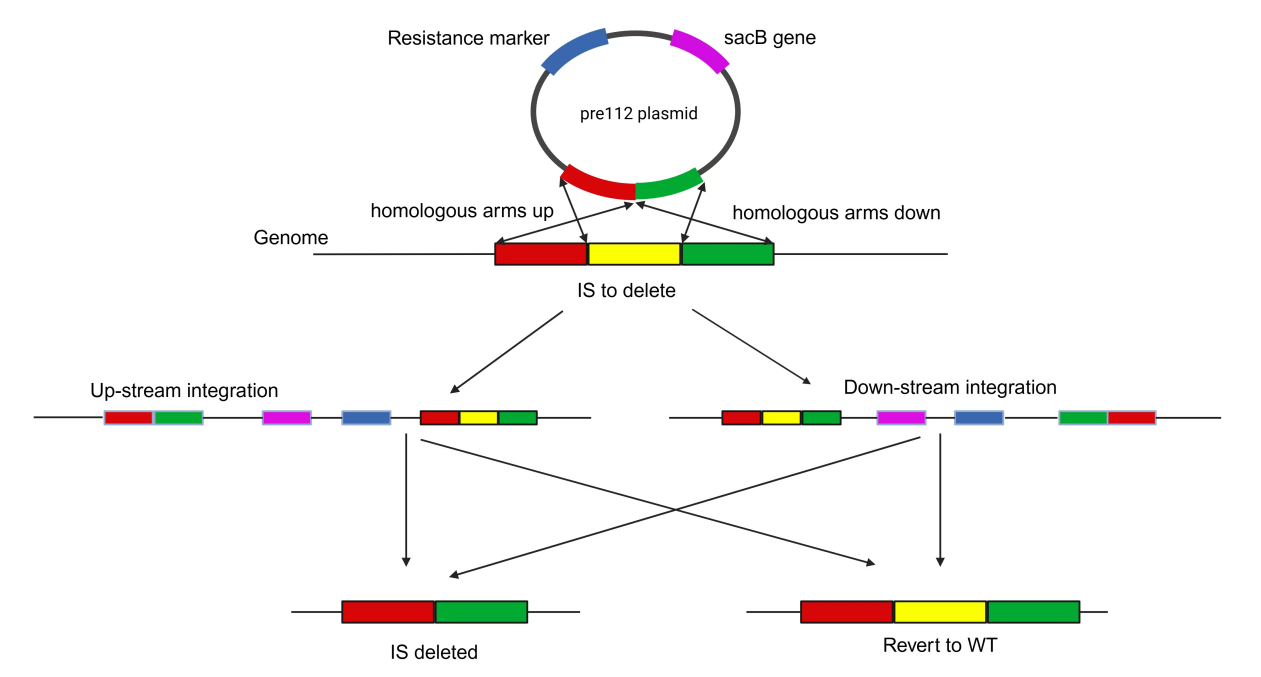


**Figure C** Schematic diagram of IS-PR knockout.

In the KP-469 strain, the suicide plasmid pRE112 was used to knock out the insertion sequence in the Ompk36 promoter region (IS-PR) [1]. For each 500 base pairs flanking the IS-PR, homologous arms were cloned into the linearized pRE112 vector. This recombinant plasmid was transformed into the competent cells of diaminopimelic acid (DAP) auxotrophic Escherichia coli strain 3064 and cultured at 37℃ overnight. A monoclonal colony was isolated and verified by Sanger sequencing. Monoclonal cultures of KP-469 and the donor strain (3064/pRE112) were grown in LB broth (chloramphenicol, 50mg/L) and 0.5 mM DAP, incubated at 37°C until OD600 reached 0.5. Cultures were plated on MH agar with DAP and incubated for 16 hours. Colonies formed on the MH agar plate were washed and resuspended in 0.9% sodium chloride. Subsequently, 50 μL of the suspension was spread onto an MH agar plate containing 50 mg/L chloramphenicol. Monoclonal colonies were then subcultured on 10% sucrose agar and incubated at 30°C. Individual colonies were selected and confirmed by Sanger sequencing to verify the successful knockout of IS-PR. A schematic diagram of the gene knockout is shown in **Figure C**.

**(D) Expression analysis of recombinant OmpK36 His-tagged fusion protein**

The Ompk36 coding sequence and its upstream promoter from KP-469 and KO-469 strains were cloned into the pBeloBAC11 vector (Beijing Zoman Biotechnology, ZK130) and transformed into BL21 (Takara, Japan). The pBeloBAC11-BL21 colonies were cultured overnight in LB medium at 37°C with 200 rpm shaking, harvested by centrifugation (4000×g, 10 min, 4°C), and lysed in Tris-HCl buffer (pH 7.4) containing 0.1 mM PMSF protease inhibitor (Beyotime Biotechnology, China) through ultrasonication (20% amplitude, 20 cycles of 10 s pulse/30 s rest). The lysate was centrifuged again, and the pellet was resuspended for SDS-PAGE. Proteins were electrophoretically transferred to PVDF membranes (0.45μm; Merck Millipore, Germany), blocked by 5% nonfat dry milk (Beyotime, China), and incubated overnight at 4°C with anti-rpoB [2,3] (1:2000) and anti-His (1:5000) antibodies (Huabio, China). The membrane was washed by TBST (tris-buffered saline, 0.1% Tween 20) and incubated with an HRP-linked antibody (Abmart, China) for 1 hour at room temperature. The membrane was finally developed by BeyoECL (Beyotime, China) and exposed to X-ray film.

**(E) The crystal structure analysis of OmpK36 in the KP-469 strain.**

(1) BLAST (Basic Local Alignment Search Tool) analysis was performed against the NCBI non-redundant nucleotide database to assess sequence homology between the OmpK36 coding sequence and documented porin genes. Significant matches were cross-validated using PSI-BLAST for conserved domain identification.

(2) Nucleotide-to-amino acid translation was executed via the ExPASy Translate Tool (ExPASy SIB Bioinformatics Resource Portal), followed by systematic domain characterization using InterProScan (v5.62-94.0) and Pfam (v35.0) databases. Porin-specific features, including β-barrel transmembrane topology and extracellular loops, were annotated based on PDB ID 5O79.

(3) The AlphaFold2 service was used to predict the three-dimensional structure of the protein. Input the translated amino acid sequence into the AlphaFold2 batch script on the Google Colab platform (https://colab.research.google.com/github/sokrypton/ColabFold/blob/main/batch/AlphaFold2_batch.ipynb) to generate a high-resolution protein model.

(4) The OPM database (Orientations of Proteins in Membranes)[4] was applied to embed the predicted three-dimensional protein structure into a lipid bilayer model, simulating its native conformation and behavior in a biological membrane environment. Subsequently, the embedded protein structure file was imported into a pore analysis tool to prepare for subsequent channel size measurements.

(5) The HOLE software (v2.2.005) [5] was used to analyze the geometric parameters of the protein pore, including the minimum pore diameter, maximum pore diameter, and the variation curve of pore size along the channel. Combine molecular dynamics simulation tools (GROMACS and AMBER) were then used to simulate the dynamic behavior of the porin protein in the membrane environment. The simulation data using the MDAnalysis library was used to analyze the temporal variation of pore size. VMD software was finally applied to visualize the pore structure, annotate the pore dimensions on the visualized model, and generate relevant analysis charts to present the results.

**
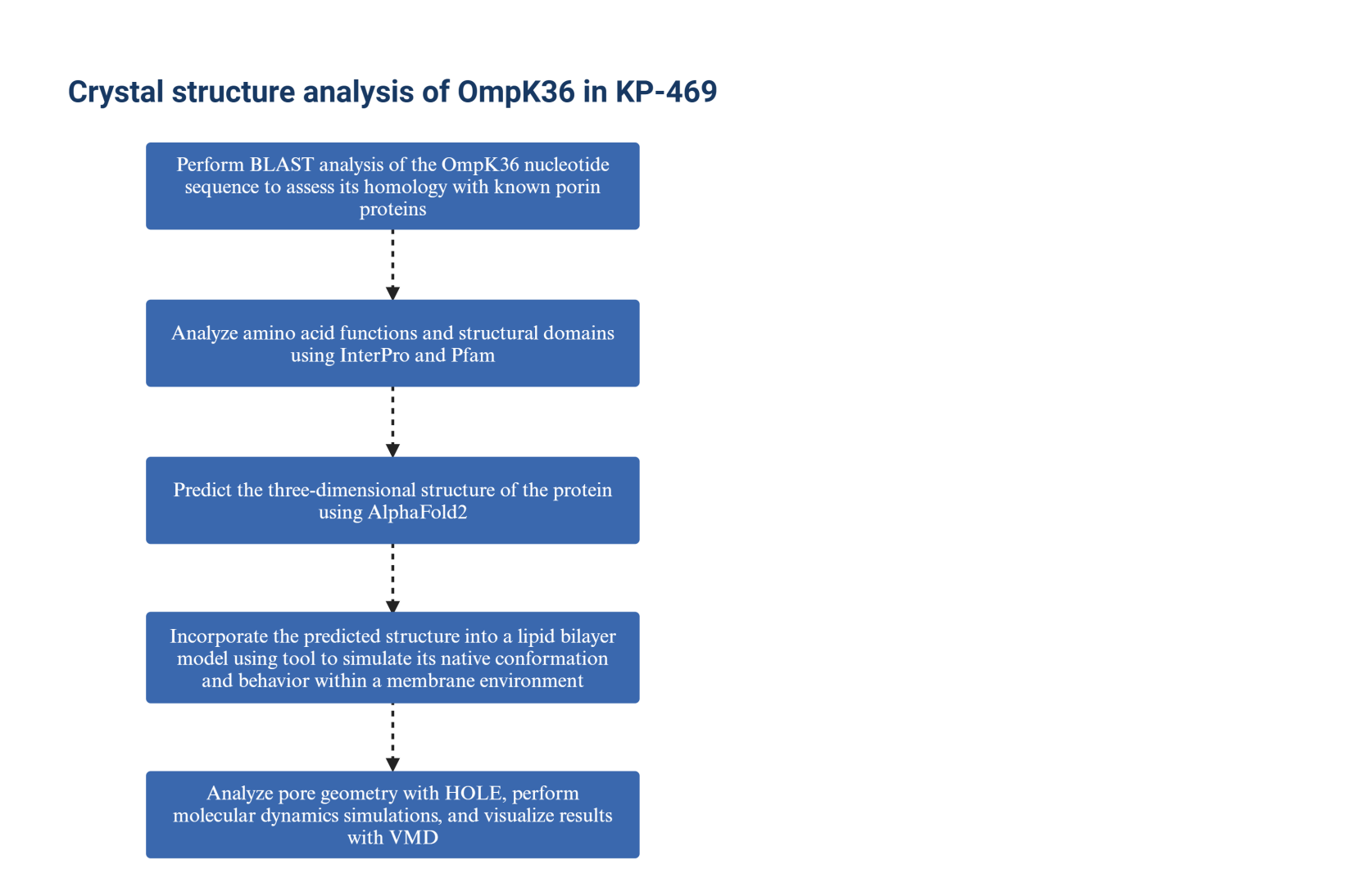
**

**Figure E Flowchart of the procedure of crystal structure analysis of OmpK36 in KP-469.**

**2. Results section**

**Figure S1**

**
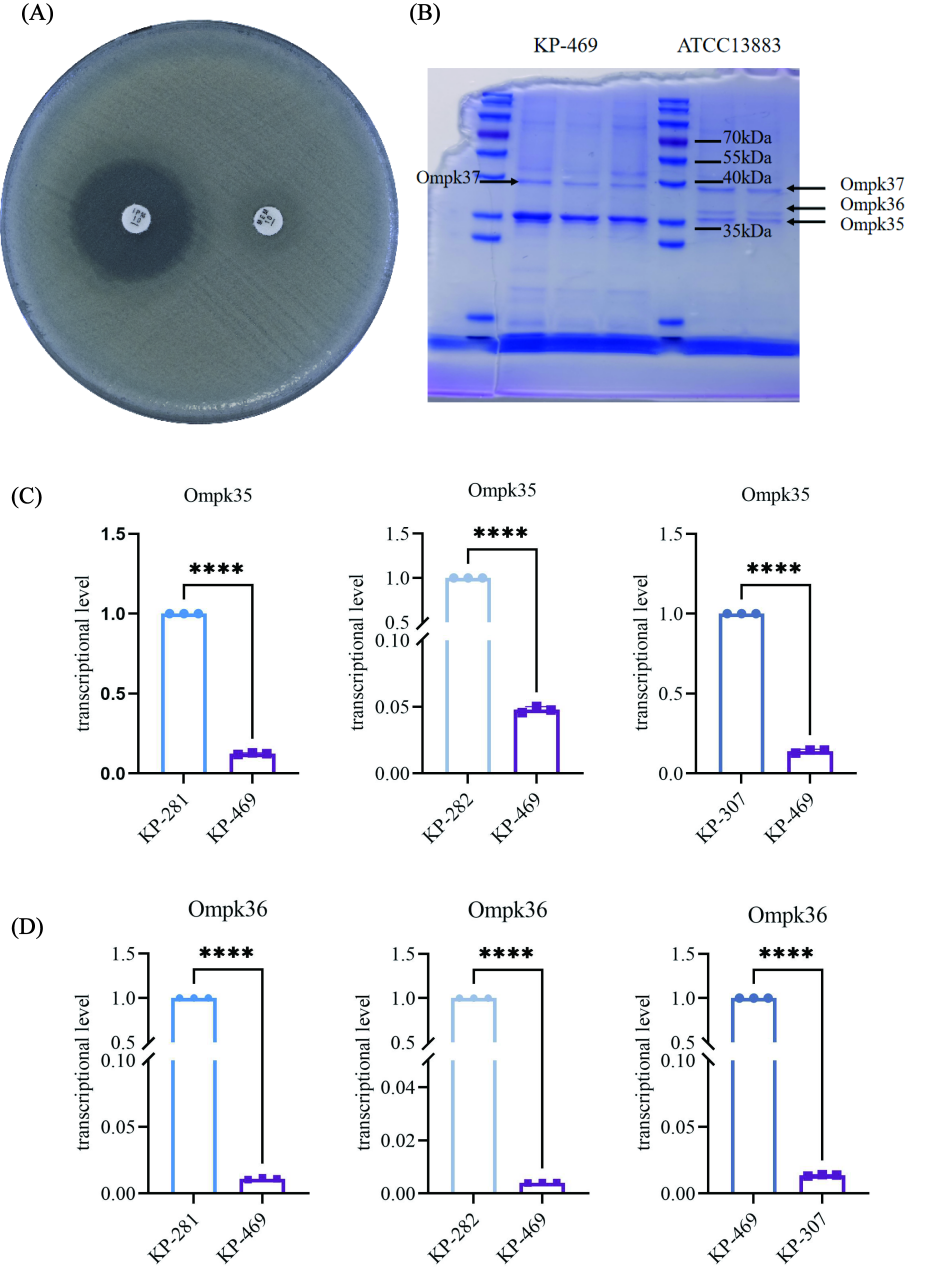
**

**Figure S1** OmpK35/OmpK36 deficiency mediated carbapenem resistance in the KP-469 strain. (A) KP-469 was susceptibility to imipenem but resistant to meropenem, as determined by the disk diffusion test. (B) SDS-PAGE found the loss of Ompk35/Ompk36 in KP-469. (C, D) The transcription levels of Ompk35/Ompk36 in KP-469 were significantly reduced, as compared with those in carbapenem-susceptible *K. pneumoniae* strains (**** represents *P* < 0.0001).

**Figure S2**


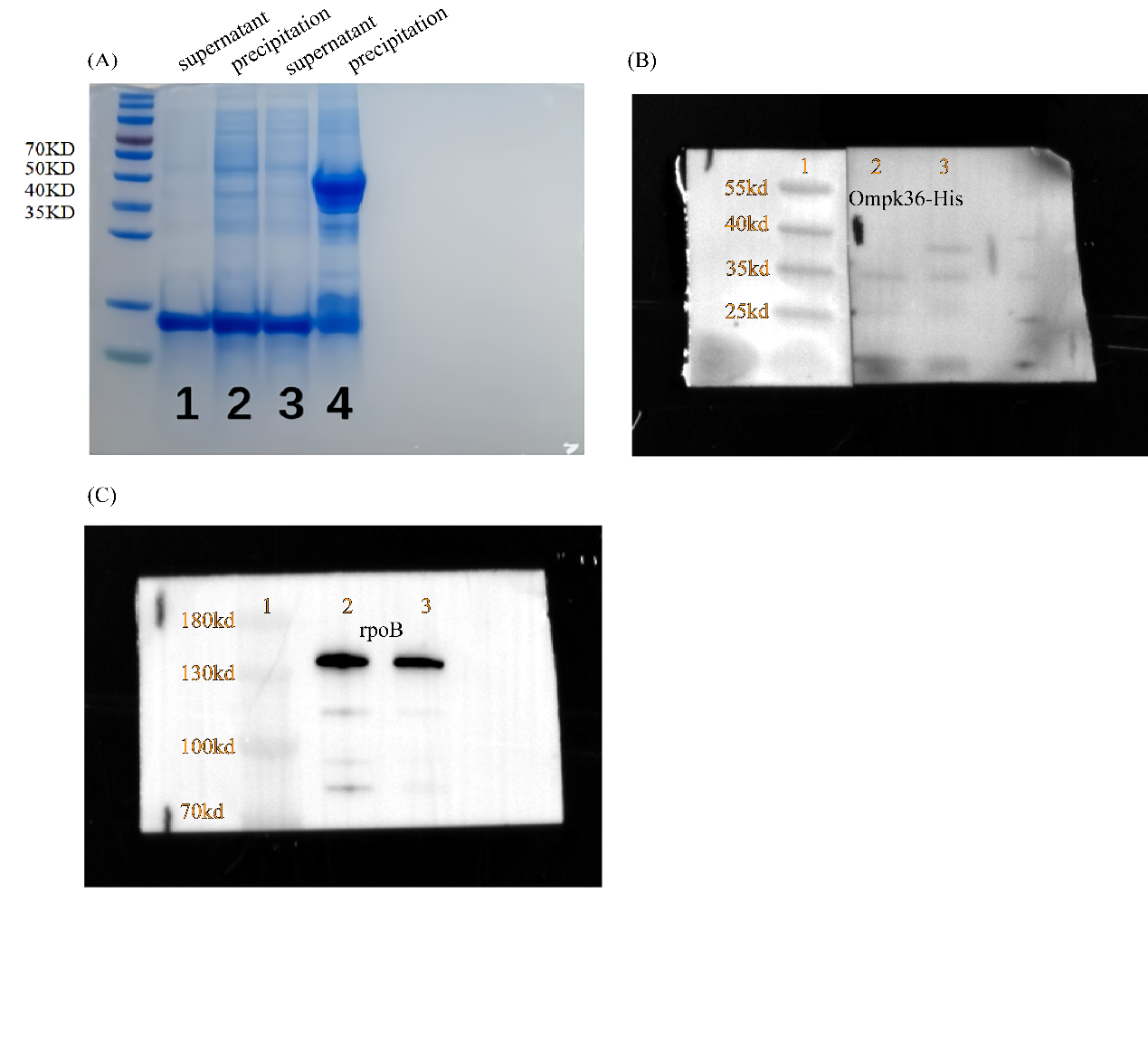


**Figure S2** IS-PR resulted in OmpK36 deficiency by translational regulation.

(A) SDS-PAGE analysis of purified OmpK36-His fusion protein. Lanes 1 (supernatant) and 2 (precipitation) represented the recombinant plasmid-transformed *E. coli* BL21 strain with the IS-PR inserted into the Ompk36 promoter; Lanes 3 (supernatant) and 4 (precipitation) represented the control strain with the wild-type Ompk36 promoter. (B) The detection of the OmpK36 His-tagged protein: 1. The protein marker; 2. The recombinant plasmid-transformed *E. coli* BL21 strain with the IS-PR interrupted promoter; 3. The control strain with the wild-type OmpK36 promoter. (C) Western blot verification using anti-OmpK36 monoclonal antibody: 1. The protein marker; 2. The recombinant plasmid-transformed *E. coli* BL21 strain with the IS-PR interrupted promoter; 3. The control strain with the wild-type OmpK36 promoter.

**Figure S3**

**
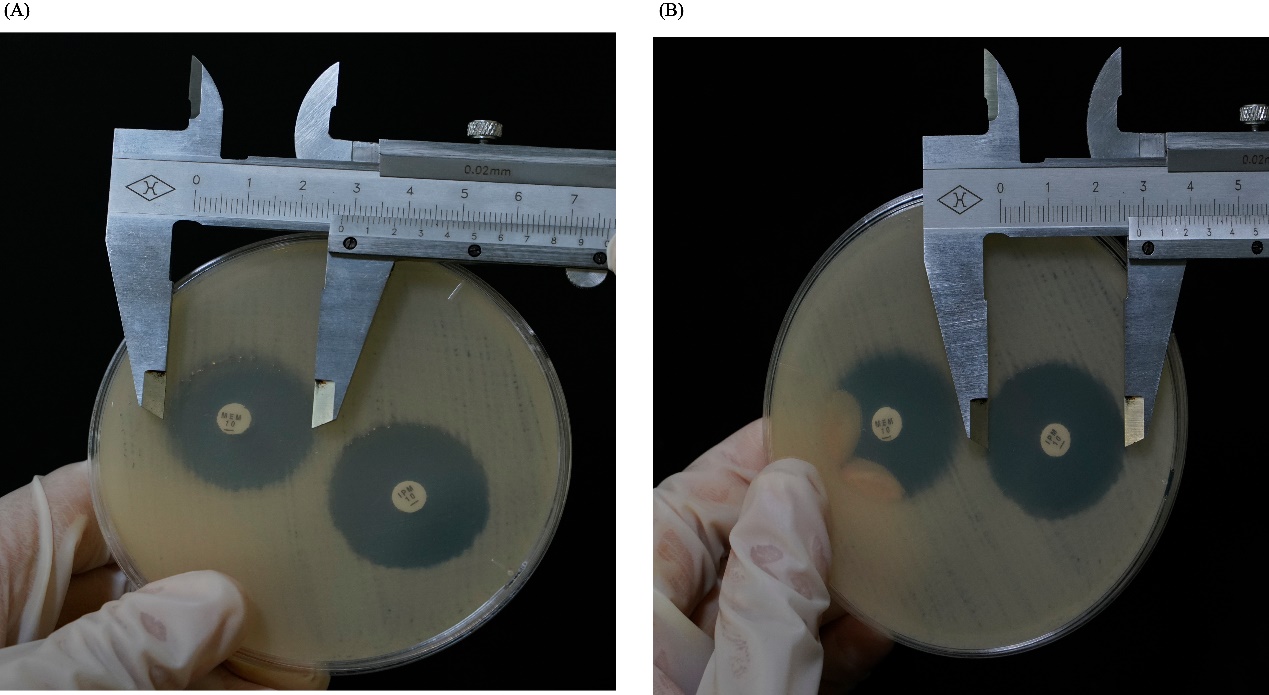
**

**
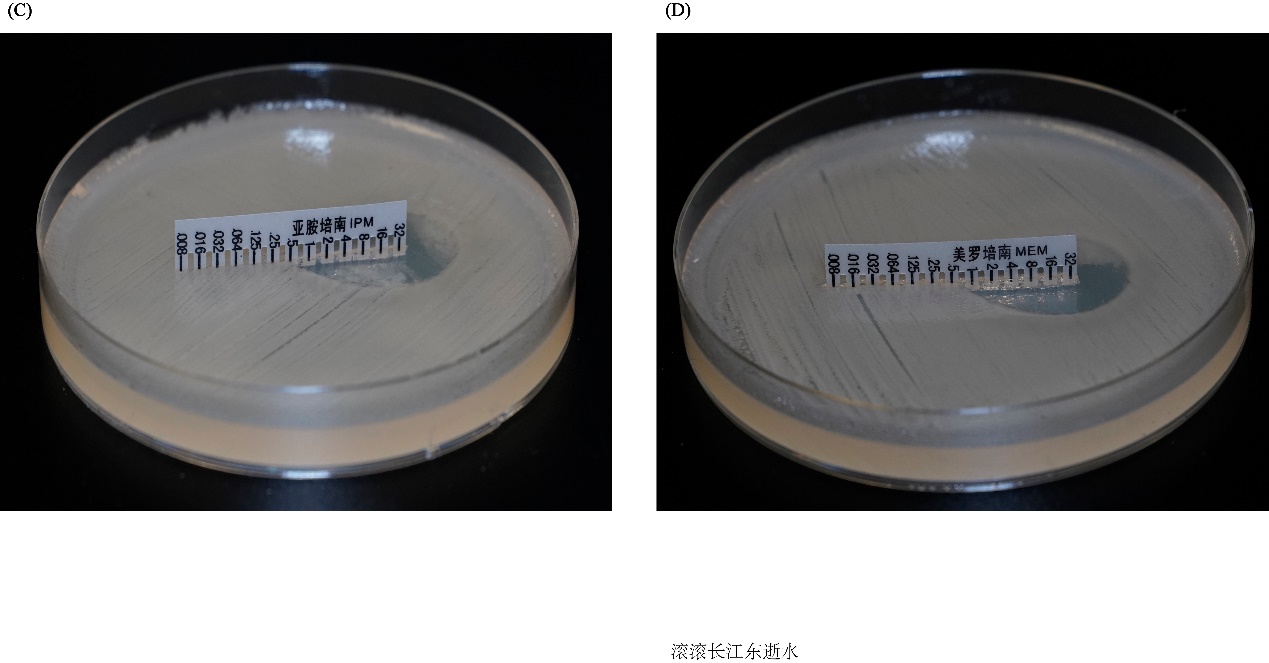
Figure S3** Both Kirby-Bauer method (A, B) and E-test strips (C, D) confirmed the restoration of carbapenem antibiotics susceptibility in KO-469. (A) The inhibition zone diameter of KO-469 against meropenem (10μg) was 26 mm. (B) The inhibition zone diameter of KO-469 against imipenem (10μg) was 27 mm. (C) The MIC of imipenem (IPM) against KO-469 was 0.5 mg/L; (D) The MIC of meropenem (MEM) against KO-469 was 1 mg/L

**Figure S4**


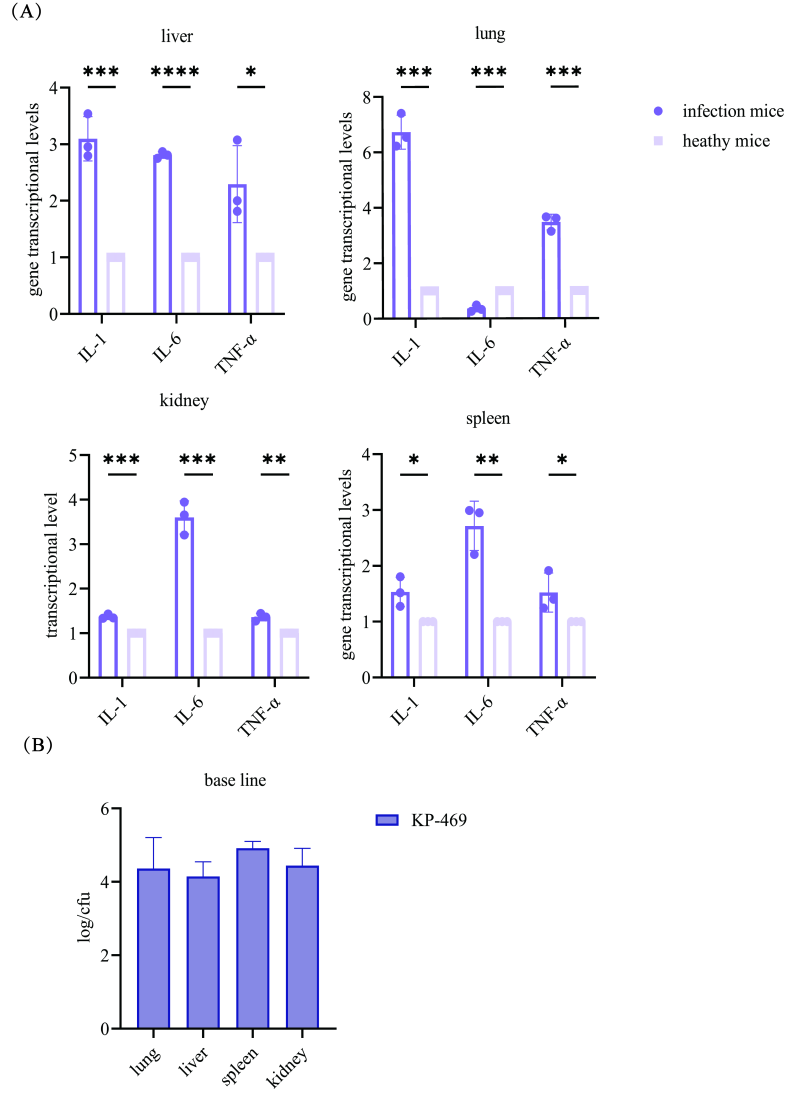


**Figure S4** Successful construction of abdominal infection model in the neutrophil-depleted mice.

(A) The levels of IL-1, IL-6 and TNF-α in mouse tissues (liver, lung, kidney and spleen) were significantly increased after intraperitoneal inoculation with the KP-469 strain, as compared to those in healthy controls (***, ** and * represent *P* < 0.001, *P* < 0.01, and *P* < 0.05, respectively). (B) The high bacterial loads in the lung, liver, spleen and kidney of the neutrophil-depleted mice after intraperitoneal inoculation of the KP-469 strain, indicating systemic dissemination of this pathogen.

1. **Reference**

[1] Zi C, Yang S, Fu X, et al. An efficient method for knocking out genes on the virulence plasmid of hypervirulent Klebsiella pneumoniae. New Microbiol. 2023 May;46(2):186-195.

[2] Huang YH, Hilal T, Loll B, et al. Structure-Based Mechanisms of a Molecular RNA Polymerase/Chaperone Machine Required for Ribosome Biosynthesis. Mol Cell. 2020 Sep 17;79(6):1024-1036.e5.

[3] <https://huabiocn/products/RNA-polymerase-beta-antibody-HA721880>.

[4] Lomize MA, Pogozheva ID, Joo H, et al. OPM database and PPM web server: resources for positioning of proteins in membranes. Nucleic Acids Res. 2012 Jan;40(Database issue):D370-6.

[5] <https://wwwholeprogramorg/>.
