# Supplementary figures and images for: Scarless excision of an insertion sequence in the OmpK36 promoter restores meropenem susceptibility in a non-carbapenemase-producing Klebsiella pneumoniae
Source: Emerg Microbes Infect. 2025 May 9;14(1):2503922. doi: 10.1080/22221751.2025.2503922 (PMC12086927; doi:10.1080/22221751.2025.2503922)

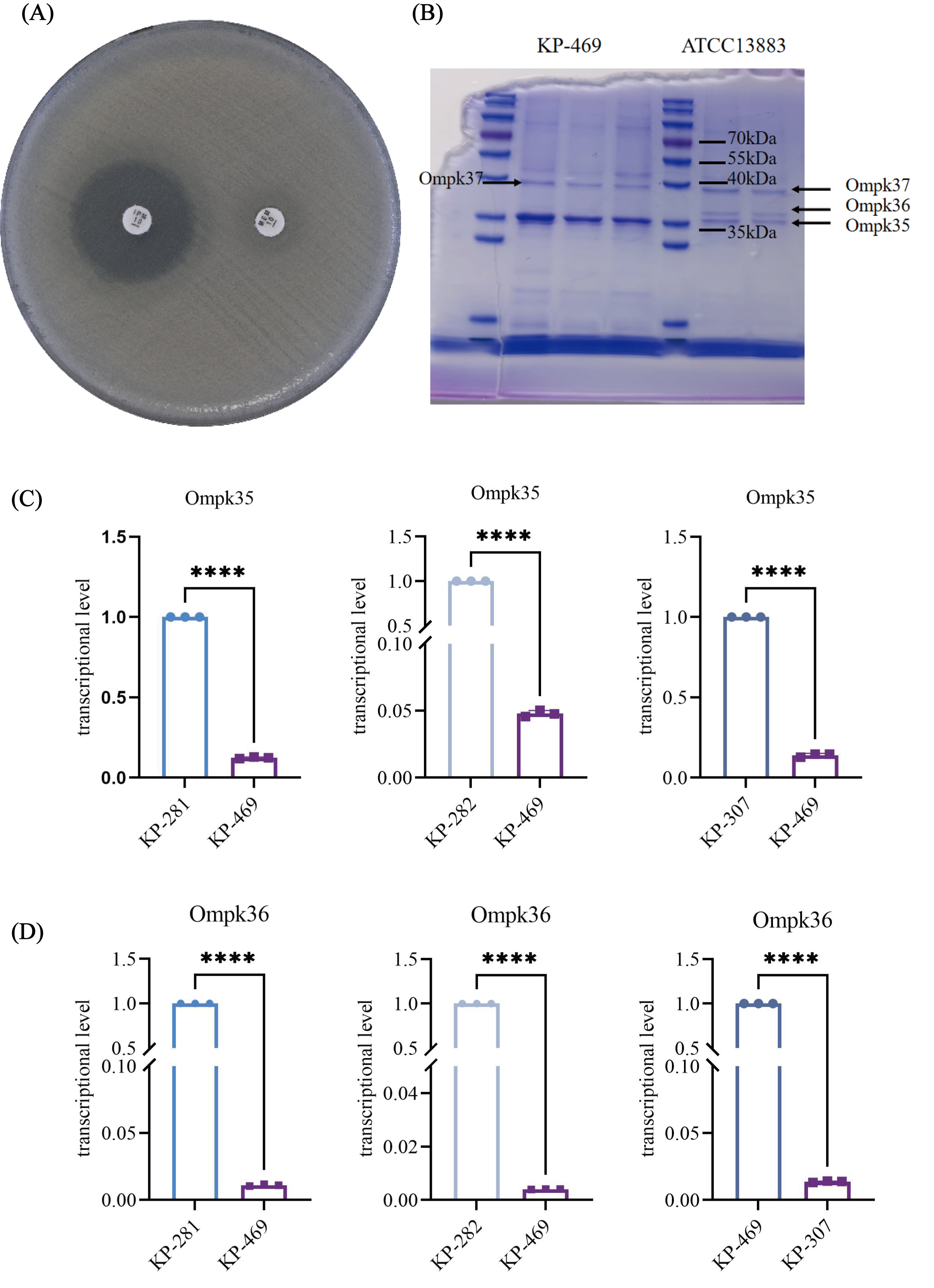

Supplement: Figure S1.tif [file TEMI_A_2503922_SM7309.tif]

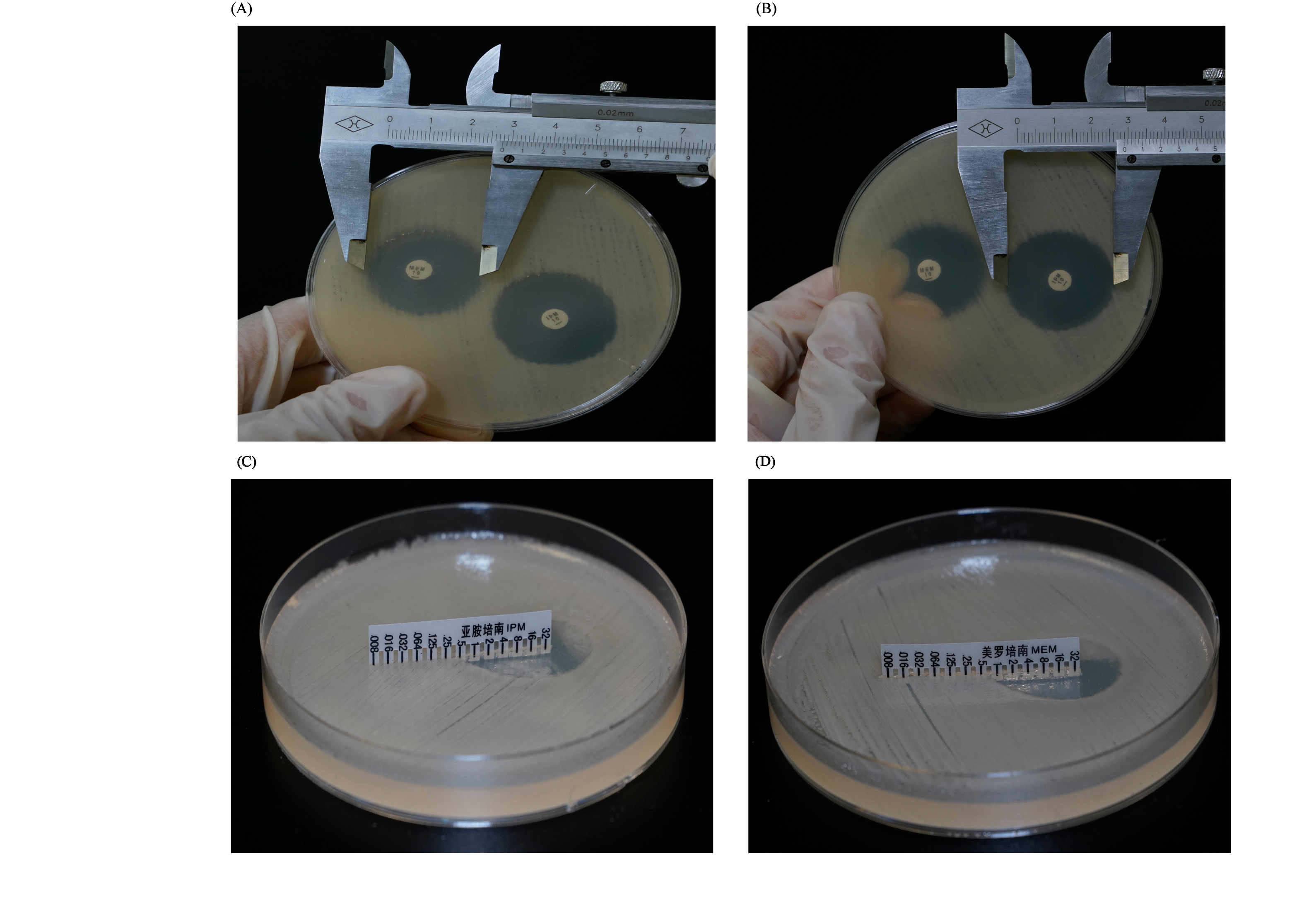

Supplement: Figure S3.tif [file TEMI_A_2503922_SM7307.tif]
